# Zero-inflated negative binomial modelling of antenatal care utilization and it’s predictors in Somaliland: *evidence from the 2020 demographic and health survey*

**DOI:** 10.3389/fgwh.2026.1763925

**Published:** 2026-03-23

**Authors:** Abdirashid Muhumed Guled, Abdulkadir Mohamed Nuh, Mohamed Abdillahi Mohamed, Ridwan A. Mohamed, Abdisalam Hassan Muse, Hamse Arab Ali, Hamse Adam Abdi

**Affiliations:** 1 Faculty of Science and Humanities, School of Postgraduate Studies and Research (SPGSR), Amoud University, Borama, Somalia; 2 Hir Institute for Research and Development, Hargeisa, Somalia; 3 School of Postgraduate Studies and Research, Gollis University, Hargeisa, Somalia; 4 College of Medicine and Health Science, University of Hargeisa, Hargeisa, Somalia

**Keywords:** ANC, pregnant woman, SLDHS, Somaliland, zero-inflated negative binomial modelling

## Abstract

**Background:**

Antenatal care (ANC) is a critical component of maternal health, directly linked to achieving global goals like Sustainable Development Goal 3. However, Somaliland faces significant challenges, with a historically high maternal mortality ratio and a high prevalence of ANC non-utilization. This study aims to determine the level and associated factors of ANC utilization, specifically accounting for the high rate of non-use, among pregnant women in Somaliland.

**Methods:**

We analyzed data from the nationally representative 2020 Somaliland Demographic and Health Survey (SLDHS). A sample of 3,152 women aged 15–49 who had a recent live birth was included the study. Given the high proportion of zero ANC visits and data overdispersion, a Zero-Inflated Negative Binomial (ZINB) regression model was employed. This method simultaneously models the factors associated with the likelihood of having any ANC visit (zero-inflated part) and the factors influencing the number of visits among users (count part). Results are presented as Incidence Rate Ratios (IRR) for the count model and Adjusted Odds Ratios (AOR) for the zero-inflated part.

**Results:**

The analysis revealed a low overall utilization rate, with 51.73% (weighted) of women reporting zero ANC visits during their last pregnancy, and a low mean of 1.7 visits. The ZINB model demonstrated a superior fit to the data. Factors significantly associated with an increased number of ANC visits (Count Part, IRR) were belonging to the Middle (IRR = 1.259), Fourth (IRR = 1.259), and Highest (IRR = 1.373) wealth quintiles. Conversely, residence in Sool (IRR = 0.786) and Sanaag (IRR = 0.732) regions, and having 3–4 children (IRR = 0.882) were associated with a reduced number of visits. Factors that significantly reduced the odds of being a non-user (Zero-Inflated Part, AOR) included primary, secondary, or higher educational attainment and mobile phone ownership (AOR = 0.425). Conversely, nomadic residence (AOR = 1.746) was associated with significantly higher odds of having zero ANC visits.

**Conclusion:**

This study demonstrates that antenatal care utilization in Somaliland remains suboptimal and reveals substantial disparities driven by regional location, nomadic lifestyle, socioeconomic status, education, parity, and access to health information. By applying a zero-inflated negative binomial model, the analysis distinguishes true structural non-users from under-users, offering deeper insight than previous approaches.

## Introduction

The provision of antenatal care (ANC) is a critical component of maternal and child health and is strongly aligned with global health and development goals. Global initiatives, such as the Millennium Development Goals (MDGs), particularly Goal 5, aimed to improve maternal health, a priority that has been carried forward with greater urgency in the Sustainable Development Goals (SDGs). Specifically, SDG 3 targets a substantial reduction in the global maternal mortality rate (1, 2). Similarly, the African Union's Agenda 2063 envisions a continent where no woman dies preventable cause related to pregnancy and child birth, underscoring the importance of full access to reproductive health services, including ANC (2–4). Adequate ANC is fundamental to achieving these targets, as it provides opportunities for health promotion, disease prevention, early detection, and management of pregnancy-related complications (1, 5, 6).

Despite global commitments, significant disparities in ANC utilisation persist, particularly in Sub-Saharan Africa. Globally, approximately 87% of pregnant women attend at least one ANC visit; however, only about 49%–53% complete the previously recommended minimum of four visits in sub-Saharan Africa (7). The situation in Somaliland is particularly alarming and requires urgent attention. The maternal mortality ratio is estimated at 396 deaths per 100,000 live births (8) and the 2020 Somaliland Demographic and Health Survey (SLDHS) reported that 62.4% of women did not receive any ANC (8, 9). This low coverage contributes to the region's high maternal mortality ratio. These poor outcomes reflect longstanding challenges within Somaliland's health system, which has been severely affected by conflict, poverty, underdevelopment, limited infrastructure, shortages of skilled health professionals, and geographic barriers, particularly for rural and nomadic populations (7, 8).

The literature on ANC utilisation has evolved from a focus on attendance counts to a broader examination of the determinants influencing the use of services. Early studies emphasised the number of visits, guided by the World Health Organization's recommendation of at least four visits, which has since been updated to eight visits to enhance care quality (10). Research across low-income and sub-Saharan African countries consistently identifies maternal education, household wealth, age, marital status, parity, employment status, and place of residence as key predictors of ANC use (11–13). Exposure to mass media, husbands’ education and support, and cultural factors also significantly influence the utilisation of ANC services (14–16). Additionally, healthcare system factors, such as the cost of services, distance to health facilities, availability of skilled providers, and perceived quality of care, play a critical role in determining ANC attendance (12, 17).

Despite increasing research on antenatal care (ANC) utilisation, Somaliland-specific evidence using advanced and distributionally appropriate statistical methods remains limited. Most existing studies rely on binary logistic regression, which oversimplifies ANC utilization and fails to account for its count-based nature, excess zero observations, and overdispersion, thereby limiting causal and policy-relevant interpretation and cannot answer the reason women have *no chance* of attending ANC. To address these limitations, this study employs a Zero-Inflated Negative Binomial regression model. This approach generates methodologically robust, policy-relevant evidence to inform targeted maternal health interventions and support progress toward SDG 3 in Somaliland. To the best of our knowledge, this study is the first to apply a zero-inflated negative binomial (ZINB) model to analyze antenatal care (ANC) utilization in Somaliland.

## Methodology

### Study area

This study was conducted in Somaliland, a self-declared state functioning as an autonomous region of Somalia in the Horn of Africa and now Israil recognized as an independent country. The region is characterized by a post-conflict context and a fragile health system, with pronounced service delivery gaps in rural and nomadic areas. These structural challenges contribute to one of the highest maternal mortality ratios globally, underscoring the critical importance of antenatal care (ANC) utilization. Data were drawn from the 2020 Somaliland Demographic and Health Survey, which provides nationally representative coverage across all six administrative regions, enabling a comprehensive assessment of ANC visit patterns.

### Study design

A quantitative, cross-sectional study design was employed, utilizing secondary data from the 2020 Somaliland Demographic and Health Survey (SLDHS). This design is appropriate for examining the prevalence of antenatal care utilization and identifying associated factors at a specific point in time. The cross-sectional nature allows for a snapshot of the population, providing valuable insights into the relationships between various predictors and the outcome variable, though it does not establish causality.

### Data source

The data for this research were sourced from the 2020 Somaliland Demographic and Health Survey, the first comprehensive, nationally representative survey of its kind in Somaliland. The SLDHS was implemented by the Somaliland Ministry of Health Development with technical assistance from ICF International through the DHS Program. This survey followed a standardized and globally recognized methodology, including the use of validated questionnaires and rigorous data collection procedures. The dataset contains detailed information on a wide range of indicators, including maternal and child health, fertility, and demographic characteristics, making it an ideal source for this analysis.

### Population and sample

**Figure 1:**
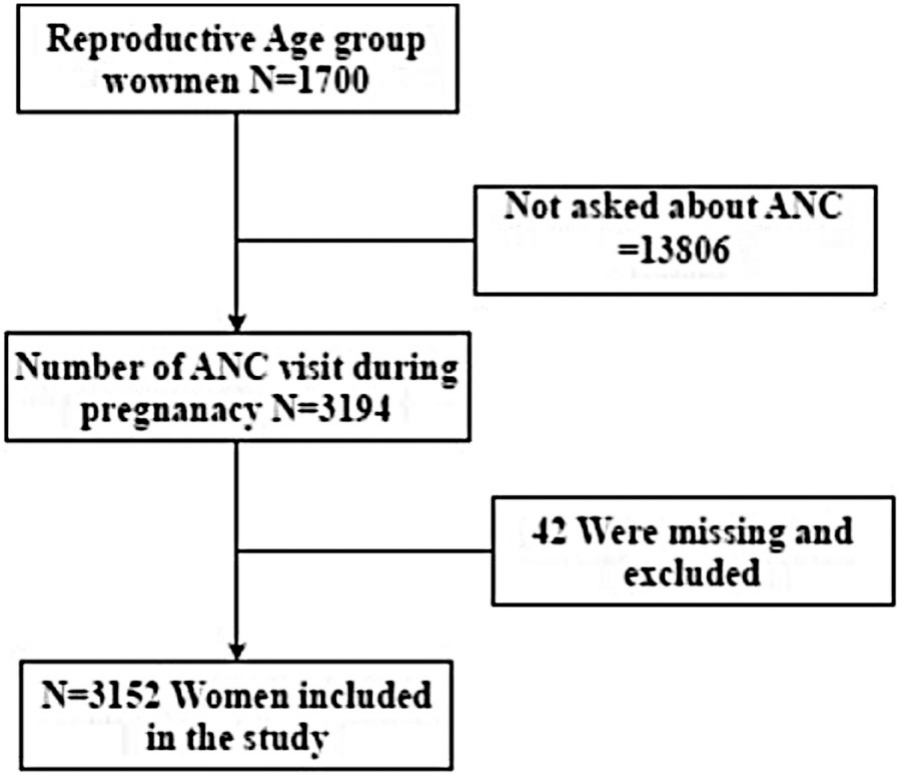
Sampling and exclusion procedures to identify the final sample size in SLDHS.2020.

The study population drawn from this survey included women of reproductive age, specifically those aged 15–49 years who had experienced at least one live birth in the five years preceding the survey. This inclusion criterion ensures that the women included in the analysis would have had a recent pregnancy and thus the potential to utilize antenatal care services. The SLDHS employed a two-stage stratified cluster sampling methodology to ensure the representativeness of the sample. In the first stage, enumeration areas were selected as the primary sampling units with a probability proportional to their size. In the second stage, households were systematically selected within each enumeration area. After excluding cases with missing data on the outcome variable or key predictors, the final analytical sample for this study was established (Figure 1).

### Study variables

The study variables were defined based on the objectives of the analysis and a review of existing literature. The primary outcome, or dependent variable, was the utilization of antenatal care, measured as a count of the total number of ANC visits a woman attended during her most recent pregnancy. This count variable ranged from zero upwards. The independent variables, or covariates, were grouped into several categories. Sociodemographic factors included the mother's age in years, her highest level of educational attainment (categorized as no education, primary, secondary, or higher), marital status, and place of residence (classified as urban, rural, or nomadic). Economic status was represented by the household wealth quintile, a composite index calculated from household assets. Obstetric and health-related variables included parity, which is the number of live births, and exposure to mass media. Media exposure was defined as exposure to at least radio, television, or newspapers at least once per week. Finally, the educational level of the woman's husband or partner was also included as a key predictor.

### Data analysis

Data analysis was conducted using Stata statistical software, version 17. The analysis began with descriptive statistics, where frequencies and percentages were used to summarize categorical variables, while means and standard deviations were calculated for continuous variables to describe the study sample's characteristics. Given that the outcome variable was count data characterized by a high proportion of zero values (women with no ANC visits) and overdispersion (variance greater than the mean), a Zero-Inflated Negative Binomial (ZINB) Poisson regression model was determined to be the most appropriate analytical approach. The ZINB model is superior to standard Poisson or logistic regression for this type of data because it simultaneously models two distinct processes: first, a logistic regression model is used to predict the factors associated with being in the “certain zero” group (non-utilization), and second, a negative binomial regression model is used to predict the number of ANC visits for those who are in the “at-risk” group (utilization). The results from the model are presented as Odds Ratios (ORs) for the zero-inflated part and Incidence Rate Ratios (IRRs) for the count part, both with 95% confidence intervals and *p*-values. The analysis accounted for the complex survey design, including sampling weights, clustering, and stratification, to produce nationally representative and statistically valid estimates.

### Sampling exclusion procedure

#### Ethical considerations

Ethical considerations for this study were addressed through adherence to established guidelines for secondary data analysis. The original 2020 SLDHS protocol was reviewed and approved by the institutional review board of the Somaliland Ministry of Health Development and the ICF Institutional Review Board. During the primary data collection, informed consent was obtained from all participants. For this secondary analysis, permission to access and use the dataset was obtained from the DHS Program. The data provided were fully anonymized and de-identified, ensuring that no individual participants could be traced and that confidentiality was maintained throughout the research process.

## Results

Table 1 presents the distribution of antenatal care (ANC) visits among women in Somaliland. More than half of the women did not receive any ANC, with 62.56% unweighted and 51.73% weighted reporting zero visits, highlighting a major gap in maternal healthcare access. Among those who sought care, utilization remained low; only 5.55% and 9.35% (weighted) had one and two visits respectively. Approximately 21% of women received three to four visits, still below the WHO-recommended minimum number of ANC contacts. Higher levels of ANC use were rare, with only small proportions attending five or more visits (ranging from 1.30% to 6.14% across categories). Overall, the mean number of ANC visits was low (1.25 unweighted; 1.7 weighted), and the standard deviation was 1.95 unweighted and 2.16 weighted, indicating substantial variability in ANC utilization among women.

**Table 1:** Number of women experiencing antenatal care utilization in Somaliland among pregnant women in Somaliland 2020.

Antenatal visits	Unweighted frequency (3,152)	Percent (%)	Weighted frequency (3,044)	Percent (%)
0	1,972	62.56	1,574.70	51.73
1	180	5.71	168.90	5.55
2	245	7.77	284.70	9.35
3	311	9.87	403.10	13.24
4	167	5.30	237.20	7.79
5	139	4.41	187.00	6.14
6	55	1.74	67.30	2.21
7	27	0.86	39.60	1.30
8+	56	1.78	81.50	2.68
Mean	**1.25**		1.7	
SD	**1.95**		**2**.**16**	

The bold values represent the summary statistics.

### Model comparison

Table 2 evaluates the performance of the Zero-Inflated Negative Binomial (ZINB) and Zero-Inflated Poisson (ZIP) models using a sample of 3,152 observations. Across all statistical criteria, the ZINB model demonstrates a superior fit. The ZINB model yields a higher log-likelihood (−4,033.954) than the ZIP model (−4,036.850), indicating a better overall fit to the data. After accounting for model complexity, the ZINB model continues to outperform the ZIP model, as evidenced by its lower Akaike Information Criterion (AIC = 8,149.908 vs. 8,155.700) and Bayesian Information Criterion (BIC = 8,398.196 vs. 8,403.988). These findings strongly suggest that the underlying count data exhibit overdispersion—where the variance exceeds the mean—a condition the ZINB model effectively accommodates through its dispersion parameter, unlike the ZIP model.

**Table 2:** Model comparison.

Model	*N*	Likelihood	ll(null)	ll(model)	*df*	AIC	BIC
ZINB	3,152	−4,033.954	−4,339.907	−4,033.954	41	8,149.908	8,398.196
ZIP	3,152	−4,036.85	−4,345.592	−4,036.85	41	8,155.7	8,403.988

### Number of antenatal care utilization of socio-demographic characteristics among pregnant women in Somaliland 2020

Table 3 shows, antenatal care (ANC) utilization in Somaliland exhibits considerable variation across key socio-demographic characteristics, as outlined in the data. Women aged 25–34 have the highest mean ANC visits (3.64 ± 1.44 unweighted; 3.68 ± 1.41 weighted), while the youngest (15–19) and oldest (45–49) age groups have minimal representation. Regional disparities are evident, with the Sanaag and Sool regions showing the highest unweighted proportions of ANC users (23.6% and 22.3%, respectively), and Marodijeh the highest when weighted (28.7%). Urban women represent 28% of the unweighted sample and 48.6% of the weighted sample, indicative of better service access. The majority of women lack formal education (78% weighted), adversely influencing ANC uptake, with educated women demonstrating better utilization patterns. Current pregnancy status reflects that 83% of respondents were not pregnant at the time of the survey, indicating recorded visits pertained mostly to past pregnancies. Media exposure is notably low, with over 92% of women not listening to the radio and 77% not watching television, limiting access to health information. Decision-making regarding healthcare is predominantly joint or husband-led (72%), suggesting limited female autonomy affecting ANC utilization. Additionally, 60% of women report distance to healthcare facilities as a significant barrier, despite high mobile phone ownership (78%). Wealth influences ANC access, with a notable shift seen in the highest wealth category in the weighted data. The average number of children ever born is 3.18 (unweighted), highlighting high fertility levels. Most respondents are married (93%), with a significant portion marrying between 12 and 18 years, which may impact reproductive health outcomes. Overall, these findings indicate that ANC utilization in Somaliland is affected by socio-economic status, education, and access barriers, underscoring the need for targeted maternal health interventions to address these disparitie.

**Table 3:** Number of antenatal care utilization of socio-demographic characteristics among pregnant women in Somaliland 2020.

Variable	Unweighted frequency			Weighted frequency		
	Number	Percent (%)	Mean ± SD	Number	Percent (%)	Mean ± SD
	Age group		3.637 ± 1.443			3.675 ± **1.406**
15–19	169	5.36		142.4	4.68	
20–24	570	18.08		508.3	16.7	
25–29	829	26.3		815.9	26.8	
30–34	674	21.38		696.2	22.87	
35–39	571	18.12		566.1	18.6	
40–44	255	8.09		239.7	7.87	
45–49	84	2.66		75.5	2.48	
	Region		13.95 ± 1.704			13.58 ± 1.635
Awdal	396	12.56		274.6	9.02	
Marodijeh	382	12.12		872.7	28.67	
Sahil	392	12.44		165.78	5.45	
Togdheer	535	16.97		792.85	26.05	
Sool	702	22.27		404.6	13.29	
Sanaag	745	23.64		533.48	17.53	
	Type residence		2.105 ± .8101			1.766 ± .8265
Urban	886	28.11		1,478.11	48.56	
Rural	1,049	33.28		798.81	26.24	
Nomadic	1,217	38.61		767.09	25.2	
	Education		.2265 ± .5549			.3131 ± .6621
No education	2,605	82.65		2,363.45	77.64	
Primary	422	13.39		475.17	15.61	
Secondary	83	2.63		138.22	4.54	
Higher	42	1.33		67.17	2.21	
	Currently pregnant		1.831 ± .3746			1.832 ± .3736
Yes	532	16.88		510.3	16.76	
No	2,620	83.12		2,533.70	83.24	
	Listen to radio		2.919 ± .3777			2.864 ± .4864
At least once a week	108	3.43		181.65	5.97	
Less than once a week	38	1.21		49.24	1.62	
Not at all	3,006	95.37		2,813.11	92.41	
	Decision on health care		2.145 ± .8170			2.148 ± .8482
Respondent	778	24.68		816.19	26.81	
Husband	1,183	37.53		1,005.90	33.05	
Jointly (respondent & husband)	1,171	37.15		1,202.29	39.5	
In-laws	3	0.1		1.82	0.06	
Someone else	9	0.29		7.7	0.25	
Other	8	0.25		10.11	0.33	
	Distance to health facility		1.339 ± .4734			1.394 ± .4888
Yes	2,083	66.09		1,842.13	60.52	
No	1,069	33.91		1,201.88	39.48	
	Watch television		2.784 ± .6022			2.579 ± .7921
At least once a week	305	9.68		583.91	19.18	
Less than once a week	69	2.19		111.99	3.68	
Not at all	2,778	88.13		2,348.10	77.14	
	Owns mobile phone		1.257 ± .4372			1.217 ± .4126
Yes	2,341	74.27		2,381.94	78.25	
No	811	25.73		662.06	21.75	
Wealth index			2.650 ± 1.567			3.157 ± 1.591
Lowest	1,160	36.8		774.16	25.43	
Second	519	16.47		406.49	13.35	
Middle	350	11.1		354.34	11.64	
Fourth	508	16.12		584.7	19.21	
Highest	615	19.51		924.3	30.36	
	Children ever born		3.181 ± .8295			3.161 ± .8431
0 child	2	0.06		1.69	0.06	
1–2 children	844	26.78		870.23	28.59	
3–4 children	886	28.11		805.92	26.48	
>5 children	1,420	45.05		1,366.17	44.88	
	Marital status		1.079 ± .3398			1.094 ± .3665
Married	2,966	94.1		2,830.14	92.97	
Divorced	120	3.81		139.79	4.59	
Widowed	66	2.09		74.08	2.43	
	Age at first marriage		1.524 ± .6052			1.562 ± .6147
12–18 years	1,684	53.43		1,533.40	50.37	
19–26 years	1,284	40.74		1,310.24	43.04	
>26 years	184	5.84		200.37	6.58	

The bold numbers indicate the mean and standard deviation (SD) of antenatal care visits for both unweighted and weighted samples.

### Factor associated with ANC utilization among pregnant women in Somaliland 2020

The factors associated with the frequency of antenatal care utilization in the Poisson model show that region, residence, wealth index and number of living children were significantly associated with ANC visit frequency (Table 4).

**Table 4:** Factor associated with ANC utilization among pregnant women in Somaliland, 2020.

Variables	Categories	IRR	*p*.value	(95% CI)	Inflated part (AOR)	*p*.value	(95% CI)
Age	15–19	1	1	1	1	1	1
	20–24	1.190	0.104	(0.965, 1.469)	−0.191	0.466	(−0.705, 0.323)
	25–29	1.221	0.073	(0.982, 1.520)	−0.097	0.724	(−0.638, 0.443)
	30–34	1.169	0.186	(0.927, 1.473)	0.012	0.967	(−0.563, 0.587)
	35–39	1.158	0.233	(0.910, 1.473)	−0.145	0.637	(−0.745, 0.455)
	40–44	1.026	0.853	(0.779, 1.352)	0.184	0.592	(−0.488, 0.856)
	45–49	1.201	0.261	(0.873, 1.652)	−0.137	0.737	(−0.939, 0.664)
Regions	Awdal	1	1	1	1	1	1
	Marodijeh	0.892	0.057	(0.792, 1.004)	0.576	0.002	(0.209, 0.943)
	Sahil	0.920	0.156	(0.821, 1.032)	0.370	0.047	(0.005, 0.736
	Togdheer	0.878	0.029	(0.781, 0.987)	0.463	0.008	(0.121, 0.805)
	Sool	0.786	<.001	(0.689, 0.897)	1.334	<.001	(0.989, 1.680)
	Sanaag	0.732	<.001	(0.646, 0.828)	1.453	<.001	(1.108, 1.797)
Residence	Urban	1					
	Rural	1.060	0.161	(0.977, 1.151)	0.739	<.001	(0.496, 0.983)
	Nomadic	0.636	<.001	(0.520, 0.778)	1.746	<.001	(1.370, 2.122)
Educational status	No education	1	1	1	1	1	1
	Primary	0.959	0.356	(0.878, 1.048)	−0.707	<.001	(−0.989, −0.425)
	Secondary	1.147	0.071	(0.988, 1.331)	−0.596	0.062	(−1.221, 0.029)
	Higher	1.092	0.37	(0.901, 1.323)	−1.202	0.059	(−2.447, 0.044)
Wealth index	Poorest	1	1	1	1	1	1
	Second	1.116	0.199	(0.944, 1.320)	0.018	0.91	(−0.303, 0.340)
	Middle	1.259	0.009	(1.059, 1.497)	0.198	0.304	(−0.179, 0.575)
	Fourth	1.259	0.006	(1.067, 1.485)	−0.409	0.031	(−0.781, −0.038
	Highest	1.373	<.001	(1.163, 1.621)	−0.593	0.003	(−0.984, 0.201)
Marital status	Never married	1	1	1	1	1	1
	Divorced	1.038	0.699	(0.860, 1.251)	0.784	0.002	(0.289, 1.279)
	Widowed	1.050	0.719	(0.804, 1.373)	0.384	0.257	(−0.280, 1.048)
Decision maker for health care	Respondent Only	1	1	1	1	1	1
	Husband	0.912	0.056	(0.830, 1.002)	−0.027	0.83	(−0.275, 0.221)
	Respondent and husband jointly	0.984	0.706	(0.902, 1.072)	−0.091	0.458	(−0.332, 0.150)
	In laws	0.569	0.336	(0.181, 1.792)			−100.325 (N/A)
	Someone else	1.115	0.691	(0.653, 1.904)	−0.748	0.385	(−2.436, 0.940)
	Other	0.512	0.127	(0.216, 1.211)	−1.434	0.341	(−4.388, 1.520)
Distance health facility	Yes	1	1	1	1	1	1
	No	1.071	0.067	(0.995, 1.152)	0.016	0.877	(−0.187, 0.219)
Currently pregnant	Yes	1	1	1	1	1	1
	No	0.996	0.933	(0.911, 1.090)	0.235	0.063	(−0.013, 0.483)
Number of living children	0	1	1	1	1	1	1
	1–2 children	0.857	0.652	(0.892, 1.243)	−10.109	0.978	(−732.7,712.5)
	3–4 children	0.882	0.013	(0.798, 0.974)	−10.050	0.978	(−732.7, 712.6)
	more than 5 children	0.892	0.058	(0.793, 1.004)	−10.06	0.978	(−732.7, 712.6)
Age at first marriage	12–18 years	1	1	1	1	1	1
	19–26 years	0.979	0.605	(0.903, 1.061)	−0.022	0.838	(−0.237, 0.193)
	Over 26 years	0.997	0.97	(0.851, 1.169)	−0.205	0.347	(−0.633, 0.222)
Frequency of listening to radio	At least once a week	1	1	1	1	1	1
	Less than once a week	1.072	0.693	(0.758, 1.516)	0.821	0.115	(−0.201, 1.844)
	Not at all	1.061	0.472	(0.903, 1.245)	0.118	0.659	(−0.407, 0.643)
Frequency of watching tv	At least once a week	1	1	1	1	1	1
	Less than once a week	1.020	0.837	(0.848, 1.226)	0.351	0.35	(−0.384, 1.085)
	Not at all	1.025	0.631	(0.926, 1.135)	0.505	0.015	(0.099, 0.912)
Owns mobile	Yes	1	1	1	1	1	1
	No	0.970	0.575	(0.873, 1.078)	0.425	<.001	(0.188, 0.662)

Regional disparities were evident, with women residing in Togdheer (IRR = 0.878, 95% CI: 0.781, 0.987), Sool (IRR = 0.786, 95% CI: 0.689, 0.897) and Sanaag (IRR = 0.732, 95% CI: 0.646, 0.828) demonstrating significantly lower ANC visit frequency compared with those in Awdal. Women in Marodijeh and Sahil also showed lower utilization, but these did not reach conventional levels of statistical significance. Residence had a marked effect on ANC frequency, where nomadic women had 36.4% fewer ANC visits (IRR = 0.636, 95% CI: 0.520, 0.778) compared with urban women. Wealth index emerged as a strong predictor, with women from middle (IRR = 1.259, 95% CI: 1.059, 1.497), fourth (IRR = 1.259, 95% CI: 1.067, 1.485) and highest (IRR = 1.373, 95% CI: 1.163, 1.621) wealth quintiles attending significantly more ANC visits than the poorest women. The number of living children showed a significant reduction in ANC frequency among women with 3–4 children (IRR = 0.882, 95% CI: 0.798, 0.974), suggesting declining ANC attendance with increasing parity.

In the zero-inflated part of the model, which identifies predictors of having zero ANC visits, several factors demonstrated strong associations. Regional differences were pronounced, where women in Marodijeh (AOR = 0.576, 95% CI: 0.209, 0.943), Sahil (AOR = 0.370, 95% CI: 0.005, 0.736) and Togdheer (AOR = 0.463, 95% CI: 0.121, 0.805) were significantly less likely to have zero ANC visits compared with women in Awdal, while women in Sool (AOR = 1.334, 95% CI: 0.989, 1.680) and Sanaag (AOR = 1.453, 95% CI: 1.108, 1.797) were more likely to have no ANC attendance. Residence had a strong effect, with rural women (AOR = 0.739, 95% CI: 0.496, 0.983) being less likely to have zero ANC visits, while nomadic women were significantly more likely to report zero ANC utilization (AOR = 1.746, 95% CI: 1.370, 2.122). Educational status was also significantly associated with zero ANC uptake; women with primary education (AOR = −0.707, 95% CI: −0.989, −0.425) were substantially less likely to have zero ANC visits, while those with secondary (AOR = −0.596, *p* = 0.062) and higher education (AOR = −1.202, *p* = 0.059) showed similarly strong but borderline significant reductions in zero utilization. Wealth index also influenced zero ANC uptake, where women in the fourth (AOR = −0.409, 95% CI: −0.781, −0.038) and highest (AOR = −0.593, 95% CI: −0.984, −0.201) wealth categories were significantly less likely to have zero ANC contact.

Marital status showed that divorced women were more likely to have zero ANC visits (AOR = 0.784, 95% CI: 0.289, 1.279) compared with never-married women. Nomadic residence and lack of mobile phone ownership also markedly increased the likelihood of having no ANC visits (AOR = 0.425, 95% CI: 0.188, 0.662).

## Discussion

Our study found that almost five out of ten women of reproductive age attended at least one antenatal care (ANC) visit. This study is lower than studies conducted in Afghanistan which indicates 69.3% (18), southern Ethiopia 76.6% (19), Zambia 69% (20), Guinea 80.3% (21), Nigeria 56.2% (22). This discrepancy may be explained by differences in health-system capacity, geographic accessibility, and the maturity of maternal health programs across countries. Somaliland continues to face structural constraints such as limited facility coverage, shortages of skilled health providers (23), and weaker referral systems, which can reduce service uptake. Sociocultural barriers, including lower awareness of ANC benefits, traditional beliefs, and limited female autonomy in healthcare decision-making, may further contribute to the lower utilization (24). How ever this study is higher than other studies conducted in Tanzania 39.3% (25), east Africa 31.1% (26), east Africa 11.16% (27), sub-Saharan Africa 38.0% (28).

The comparatively higher level observed in Somaliland may reflect improvements in health-service accessibility over recent years, including expansion of primary health facilities, increased deployment of skilled birth attendants, and strengthened community-based maternal health initiatives supported by governmental and non-governmental partners. Differences in study periods, measurement approaches, and population characteristics may also contribute to the variation.

Regional differences in ANC use were pronounced in this study, with significantly lower ANC utilization and higher odds of being a structural non-user observed in Togdheer, Sool, and Sanaag (27). Recent analyses from Somaliland and Somalia also show persistent geographic inequities in maternal healthcare, with women in remote regions facing limited health facility availability, insecurity, and difficult terrain (29). A 2024 Somaliland study similarly identified Sool and Sanaag as the lowest-performing regions in ANC completion, confirming that regional disparities remain one of the strongest and most consistent predictors of ANC use. Multi-country DHS reviews published in 2023–2025 further emphasize that regional location is one of the primary determinants of zero-ANC use in low-infrastructure settings (30).

The present findings also showed that nomadic women had substantially lower ANC utilization and significantly higher zero-ANC probability, while rural women had higher zero-use but nonsignificant count differences (30). This pattern aligns closely with contemporary studies on pastoralist and nomadic populations in Somalia, Ethiopia, and Kenya, which repeatedly show that mobility, long distances, and lack of fixed health facilities severely reduce maternal health service use (31). A 2025 preprint analyzing nomadic maternal health in East Africa reported that nomadic status was among the strongest predictors of non-use of ANC, supporting the conclusion that conventional facility-based models are insufficient for these population (32).

Educational status was not significantly associated with the number of ANC visits but strongly influenced the likelihood of any ANC use, with primary and secondary education reducing the odds of being a structural non-user (33). This pattern mirrors findings from recent systematic reviews showing that women's education is more strongly associated with the probability of entering the health system than with visit frequency once care is initiated (33, 34). Recent studies from Ethiopia, Nigeria, and Somalia confirm that education acts primarily through increasing awareness of pregnancy risks, navigating the health system, and improving autonomy in care-seeking, which is consistent with the present results (35).

Wealth index emerged as one of the strongest and most consistent predictors, with middle, fourth, and highest wealth categories associated with significantly higher ANC visit frequency and lower zero-ANC use. This finding is strongly supported by multiple recent DHS-based analyses published between 2022 and 2025, which consistently report that household wealth predicts both ANC initiation and the number of visits completed (33). Wealth is repeatedly highlighted as a key determinant due to indirect costs such as transportation, childcare needs, and opportunity costs that disproportionately affect poorer women. The strong dose–response pattern observed in this study is therefore fully aligned with the broader literature.

Marital status showed limited impact on visit frequency but divorced women had significantly higher odds of being zero-users, which is consistent with studies from Nigeria, Tanzania, and Ethiopia reporting that divorced or single women often face financial vulnerability, social isolation, and lack of partner support that restrict access to maternal care (36). Recent qualitative research in Somali communities also documents stigma and limited decision-making support for women without partners, which may explain this pattern.

Household decision-making did not show a clear effect in the fully adjusted model, which is consistent with emerging research indicating that autonomy effects may diminish after adjusting for wealth and education (37). However, the general direction of effects observed in this analysis, along with recent studies from East Africa, still suggests that limited autonomy may reduce likelihood of any ANC use, especially when decision-making power rests with in-laws or other household members. Parity showed a clear association in the count model, with women having three to four children exhibiting significantly fewer ANC visits compared to first-time mothers. This aligns with several 2023–2025 studies showing that higher-parity women are less likely to seek ANC early or complete recommended contacts due to increased confidence from previous births, competing responsibilities, or perceived normality of pregnancy (38). High-parity women are consistently identified as a group with reduced ANC engagement in the literature.

Media exposure and mobile phone ownership were important in predicting ANC zero-use in this analysis (39) Watching TV at least once per week and owning a mobile phone significantly reduced the probability of being an ANC non-user. These findings are similar to recent evidence from mHealth and media-exposure studies conducted in low-resource settings, which show that exposure to maternal health information through TV, radio, or mobile platforms increases awareness, timely initiation, and ANC attendance (21, 40–46). A digital health intervention concluded that mobile-phone-based reminder systems and health messaging significantly improve ANC uptake, supporting the relevance of digital strategies in Somaliland.

## Conclusion

This study demonstrates that antenatal care utilization in Somaliland remains suboptimal and reveals substantial disparities driven by regional location, nomadic lifestyle, socioeconomic status, education, parity, and access to health information. By applying a zero-inflated negative binomial model, the analysis distinguishes true structural non-users from under-users, offering deeper insight than previous approaches. The findings underscore the need to prioritize underserved regions such as Sool, Sanaag, and Togdheer; expand mobile and community-based services for nomadic groups; strengthen the health system through improved facility coverage, skilled workforce distribution, and referral networks; and enhance information access through media, mHealth, and educational initiatives. Targeted support for vulnerable groups including divorced women, high-parity mothers, and poorer households is essential to reduce inequities. Collectively, these strategies can accelerate progress toward equitable and universal ANC coverage in Somaliland.

## Data Availability

The original contributions presented in the study are included in the article/Supplementary Material, further inquiries can be directed to the corresponding author.
